# Minute Pulmonary Meningothelial-Like Nodules: An Incidental Benign Entity in Association With Lung Adenocarcinoma

**DOI:** 10.1155/crra/3311702

**Published:** 2025-04-10

**Authors:** Selima Siala, Nabil Rahoui, Frederic Askin, James F. Gruden

**Affiliations:** ^1^Department of Radiology, The University of North Carolina at Chapel Hill, Chapel Hill, North Carolina, USA; ^2^Department of Pathology and Laboratory Medicine, The University of North Carolina at Chapel Hill, Chapel Hill, North Carolina, USA

## Abstract

We report a case of a 69-year-old female smoker who presented with multiple pulmonary micronodules incidentally noted during imaging for aortoiliac occlusive disease. A growing right lower lobe nodule was resected, revealing adenocarcinoma alongside benign minute pulmonary meningothelial-like nodules (MPMNs). MPMNs, often found incidentally, in association with malignancies, can mimic metastatic disease but are benign and stable. Recognizing MPMNs is essential to prevent misdiagnosis and unnecessary treatment in patients with coexisting malignancies. On chest CT, these nodules are typically peripherally located along the interlobular septa, consistent with their close vascular association around the small pulmonary veins observed in pathology.

## 1. Introduction

Minute pulmonary meningothelial-like nodules (MPMNs) are often incidental findings in lung imaging or surgical specimens, particularly in patients undergoing evaluation for other conditions [1]. Although benign and typically stable, MPMNs can resemble metastatic nodules, especially in individuals with existing malignancies such as pulmonary adenocarcinoma [2]. This similarity poses a diagnostic challenge, potentially leading to misdiagnosis and unnecessary treatment. This manuscript presents a case of a 69-year-old female smoker with coexisting adenocarcinoma and MPMNs, highlighting characteristic radiological findings on high-resolution chest CT as well as the importance of accurate pathological evaluation to differentiate benign MPMNs from metastatic disease and ensure appropriate clinical management.

## 2. Case Report

**Figure 1:**
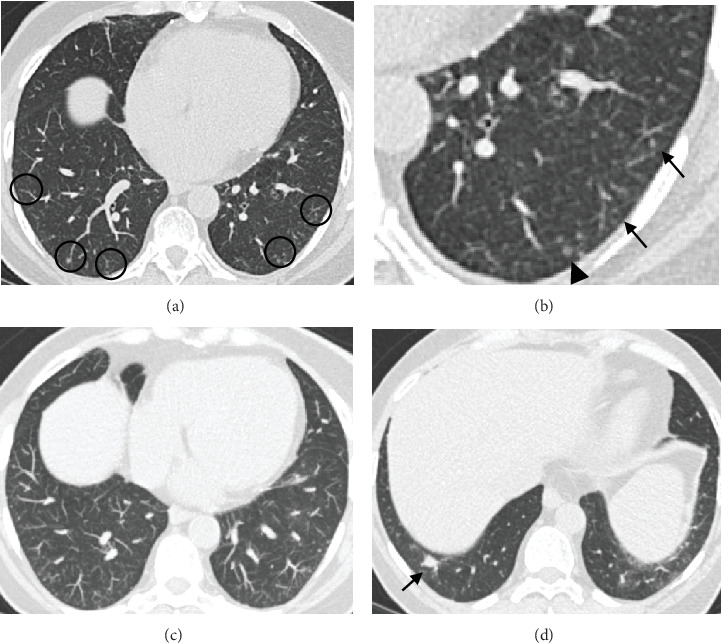
(a) Axial noncontrast chest CT (1-mm slice thickness) image shows innumerable bilateral tiny nodules of varying size, shape, and densities, predominantly in clusters in the lung bases and near the pleural surfaces (circles). (b) Axial image zoomed in the left lower lobe better demonstrates the nodules with varying size and densities (arrows), one of which has central lucency (arrowhead). (c) Axial image reconstructed with a 3-mm slice thickness using maximum intensity projection (MIP) shows distribution of the nodules in clusters near the pleural surface, sparing the pleura. (d) Axial image at the lung bases shows the dominant 1 cm solid pulmonary nodule in the right lower lobe (arrow).

**Figure 2:**
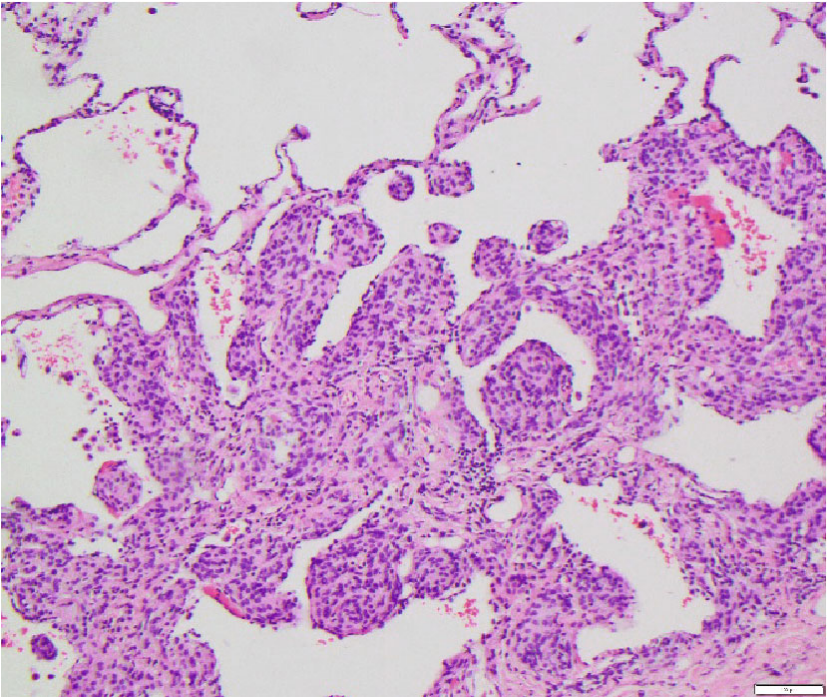
Resection specimen on hematoxylin and eosin staining (100×) shows nests of bland spindled cells arranged in a whorled pattern.

**Figure 3:**
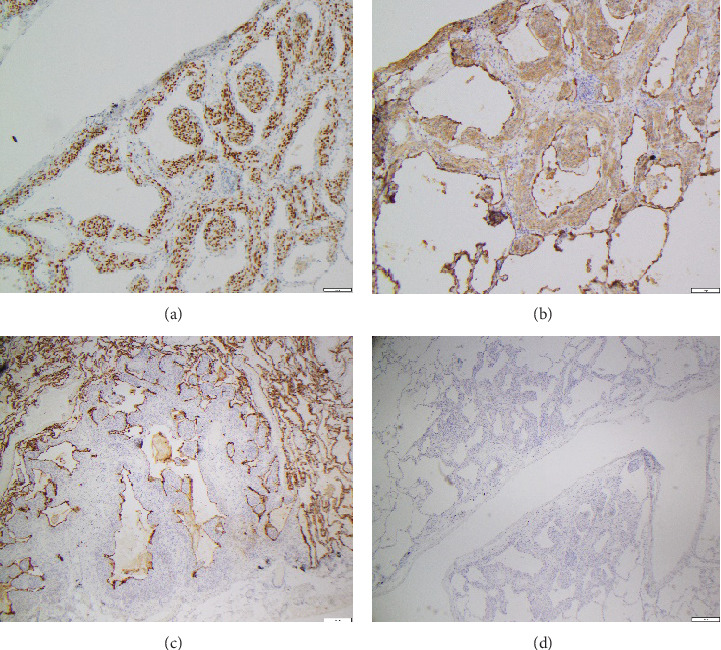
Immunohistochemistry of the resection specimen (40×). (a) Strong nuclear staining for PR. (b) Patchy positive cytoplasmic and membranous staining for EMA. (c) Negative for pancytokeratins (AE1/AE3). (d) Negative for synaptophysin.

A 69-year-old female current cigarette smoker with a 30 pack-year smoking history, hypertension, and coronary artery disease with remote myocardial infarction and coronary artery bypass grafting presented with multiple pulmonary micronodules (measuring under 5 mm) incidentally detected on a computed tomography angiography (CTA) of the abdomen performed to evaluate aortoiliac occlusive disease. A noncontrast chest CT performed 6 months later showed increased size of a dominant right lower lobe nodule and numerous bilateral stable micronodules (Figure 1). A later comparison with remote chest CTs from 2 to 5 years earlier confirmed the long-term stability of the micronodules. Given the suspicion of primary lung malignancy in a smoker with a growing right lower lobe nodule, the patient underwent a right lower lobectomy. Histological examination confirmed a moderately differentiated adenocarcinoma in the right lower lobe measuring 1 cm and innumerable unrelated pulmonary micronodules measuring 2–4 mm, consisting of nests of bland spindled cells arranged in a whorled pattern (Figure 2). On immunohistochemistry, these nodules were diffusely positive for CD56, progesterone receptor (PR), and epithelial membrane antigen (EMA) and negative for synaptophysin and cytokeratin, consistent with the diagnosis of MPMNs (Figure 3).

## 3. Discussion

MPMNs are a distinct form of asymptomatic interstitial cell proliferation. The exact etiology remains elusive, but prevailing evidence suggests that these lesions represent benign reactive proliferation rather than a neoplastic process, and do not require a specific treatment [1]. MPMNs are most common in the sixth decade and have a notable female preponderance with a female-to-male ratio reaching 12:1 [2]. This disparity may be related to the frequent expression of PRs within MPMN tissues [3]. Because MPMNs are asymptomatic, they are most often incidental findings during autopsies or surgical lung resections performed for unrelated reasons, as well as during video-assisted thoracic surgeries (VATS) or percutaneous biopsies usually performed to assess for suspected metastases in patients with known malignancy [1, 2, 4]. The advent of high-resolution CT (HRCT) scanning, particularly the utilization of thin-slice techniques for early disease detection and management, has led to an increased identification of incidental pulmonary nodules, MPMNs included [5]. It has been observed that MPMNs are frequently associated with lung cancers, particularly showing a higher incidence in conjunction with pulmonary adenocarcinoma [2, 3]. This association might stem from a more frequent use of serial imaging in these patients, suggesting that MPMNs could be more common than previously thought. Moreover, Li et al. highlighted that the primary surgical intervention in the majority of MPMN cases was motivated by the presence of malignant nodules rather than the MPMNs themselves, making their removal an incidental occurrence primarily in patients diagnosed with lung adenocarcinoma [1]. Additionally, the coexistence of MPMN with primary pulmonary meningioma (PPM) has been reported, and these entities, although both considered pleuropulmonary meningothelial proliferation, can be distinguished based on pathology features [6]. The occurrence of MPMNs appears to be unrelated to smoking or occupational exposures [7]. Clinical evaluation of lung function, tumor markers, and immune indicators typically yields normal results in patients with MPMNs, aiding clinicians in excluding other pulmonary conditions [1, 8].

Originally identified by Korn et al. in 1960, MPMNs are clusters of epithelioid cells, often the “zellballen” pattern characteristic of paraganglioma (chemodectoma) and strategically positioned around small veins in the interlobular pulmonary septa. The structures were initially thought to be neuroendocrine cells due to their architecture, cytologic features, and close vascular associations and were postulated to serve as chemoreceptors monitoring oxygen levels, leading to their initial designation as “pulmonary chemodectomas” [2, 9]. Although the histogenesis of MPMN remains unclear, subsequent research has generally suggested that MPMNs are of meningothelial derivation [3, 10, 11]. Kuhn and Askin and subsequently others found that by ultrastructural examinations, MPMN cells lack endocrine granules, are not associated with nerve structures, and closely resemble meningothelial cells [10, 12, 13]. This insight prompted the reclassification of these nodules to “minute meningothelial-like nodules” by Gaffey et al. in 1988 [10]. Alternatively, others have demonstrated the nodules' immunopositivity for myosin and vimentin, suggesting a muscular origin [3]. Connections between MPMN and central nervous system meningiomas have been suggested based on shared genetic pathways [11]. However, contrasting reports by Ionescu pointed out the absence of mutational characteristics common in meningiomas, advocating for a reactive rather than neoplastic interpretation of MPMNs [14].

Genetic analyses have generally aligned MPMNs with benign, reactive proliferation process [1, 7]. However, the discovery of increased genetic instability in cases of diffuse pulmonary meningotheliomatosis (DPM) suggests a potential intermediary phase between reactive and neoplastic states [1, 3, 14]. Earlier research indicates that the frequency of MPMN identification through autopsies ranges from 0.07% to 4.9% [2], but this rate can increase more than 10-fold when pathologists actively look for these lesions [12]. In comparison, the detection rate in surgical specimens is significantly higher, between 7% and 13.8% [2], surpassing the rates found in autopsies. This underscores the possibility that the true prevalence of MPMNs may exceed current estimates. It is essential to differentiate MPMNs from pulmonary carcinoid “tumorlets” on a histological basis. Carcinoid tumorlets are aggregates of neurosecretory cells measuring less than 0.5 cm, usually located near terminal bronchioles, and might be mistaken for meningothelial nodules [15]. Anatomically, the two lesions differ in location, and cytologically, the cells within tumorlets exhibit higher nuclear–cytoplasmic ratios, have a more stippled chromatin texture, and are spindled rather than epithelioid in appearance. PMN cells display a more epithelioid appearance with nests of oval, round cells with eosinophilic cytoplasm, and indistinct cell borders that may contain intranuclear cytoplasmic inclusions [16]. Immunohistochemical analysis further differentiates these entities, with tumorlets displaying weak cytoplasmic cytokeratin staining and positivity for neuroendocrine markers such as chromogranin, synaptophysin, and neuron-specific enolase, thereby facilitating their distinction from meningothelial-like nodules which are characterized by immunopositivity for PR, EMA, and Somatostatin Receptor 2a (SSTR2A) [17]. Note that, similar to meningiomas, MMPNs are often positive for CD56, and that antibody should not be used to separate MPMNs from carcinoid tumorlets.

It is also important to distinguish MPMNs from other pleuropulmonary meningothelial proliferations including PPMs and metastatic pulmonary meningioma, as these share similar morphological and immunohistochemical features. MPMN can be distinguished from PPM by their smaller size, perivenular pattern of growth, and ill-defined borders [10, 18].

On chest CT scans, MPMNs manifest as isolated pulmonary nodules and can be discovered incidentally on imaging performed due to other indications [8]. They can present as a single lesion or scattered nodules and can also manifest as multiple diffuse small lung nodules, also referred to as DPM [8]. While certain radiological features suggestive of MPMNs have been described, these remain nonspecific and are sometimes difficult to distinguish from atypical adenomatous hyperplasia (AAH) or metastatic disease, particularly in patients with existing malignancies. The stability of these lesions over time underscores their benign nature emphasizing the need for cautious evaluation in patients with coexisting malignancies, to avoid misinterpreting these nodules as metastatic without further pathologic correlation, potentially compromising curative surgical interventions. Characteristic CT findings of MPMNs include small round nodules typically around 5 mm in diameter with smooth margins and ground glass attenuation occasionally exhibiting central lucency or cystic-like features [19, 20]. These nodules, especially when multiple, tend to be randomly distributed but frequently located in clusters near pleural surfaces [19]. Due to the low specificity of these imaging features, distinguishing them from malignant pulmonary nodules based solely on single-image examinations is challenging. However, according to reports of previous studies, the lack of growth in MPMNs over documented follow-up periods of at least 6 months suggests that such nodules, if not changing over time or in response to systemic antitumor treatments, should be considered potential MPMNs rather than metastases [21].

**Figure 4:**
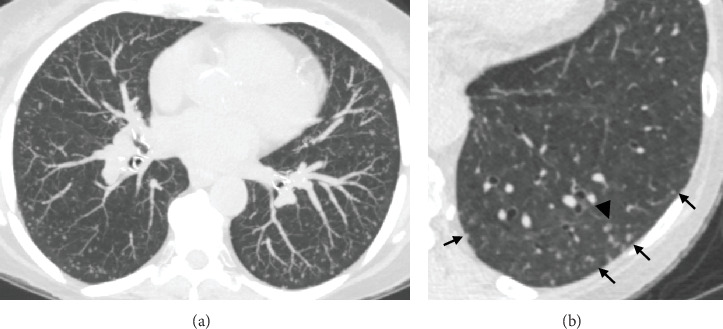
Companion case of multiple stable micronodules, presumed DPM, in a 65-year-old female. (a) Axial noncontrast chest CT image reconstructed with a 5-mm slice thickness using MIP shows bilateral innumerable and diffuse micronodules in clusters predominantly near the pleural surfaces. (b) Axial thin slice image (0.6-mm slice thickness) zoomed in the left lower lobe shows the micronodules with variable size, shape, and densities (arrows), one of which with central lucency (arrowhead).

In our experience, we noticed a characteristic peripheral location of the meningothelial nodules, sparing the pleural and fissural surfaces which distinguish them from intrapulmonary lymph node that can have a similar morphology. These nodules are often located along the interlobular septa, which further supports the close vascular association around the small pulmonary veins on pathology description. Additionally, an important feature of MPMN is the heterogeneity of density and shape of the nodules, similar to nodules of pulmonary Langerhans cell histiocytosis (LCH), with usually association of small solid, ground glass and cystic nodules in a same patient. A companion case of a presumed DPM is illustrated in Figure 4, demonstrating characteristic CT findings of multiple MPMNs. Differential diagnosis of MPMNs on HRCT scans include pulmonary metastasis, AAH, hypersensitivity pneumonitis, and LCH as mentioned above, particularly when nodules display central lucency. Notably, LCH and hypersensitivity pneumonitis primarily affect the upper lobes and are associated with a smoking history in LCH patients and antigen exposure in those with hypersensitivity pneumonitis.

## 4. Conclusion

MPMNs, first identified by Korn et al. as chemoreceptors due to their morphology and vascular proximity, have since been aligned with meningothelial cells, though their exact origin remains uncertain. The widespread use of chest HRCT has amplified the identification of incidental anomalies, including MPMNs, emphasizing the need for clinician awareness to guide management effectively. Typically asymptomatic, MPMNs are managed conservatively with surveillance imaging, highlighting the importance of recognizing their imaging characteristics, especially that this entity is frequently encountered in association with malignancies. MPMNs, often detected incidentally in surgical specimens or on chest CT scans, present as isolated or multiple small nodules usually in clusters near pleural surfaces and interlobular septa and may appear solid, ground-glass, or cystic-like. Their radiological and histological characteristics suggest an interstitial lung origin, necessitating careful differential diagnosis, particularly considering pulmonary metastasis in patients with known malignancies. While research into MPMNs and DPM continues, current evidence suggests a benign, reactive nature, guiding a primarily observational management approach.

## Data Availability

Data sharing is not applicable to this article as no datasets were generated or analyzed during the current study.
